# *Analytical Science Advances* announces its new Associate Editorial Board

**DOI:** 10.1002/ansa.202100901

**Published:** 2022-01-06

**Authors:** Paul Trevorrow, Daniel Petras, Lissa C. Anderson, Tal Luzzatto Knaan, Anthony J. Saviola, Ignacy Rzagalinski, Mohammad Sharif Khan, Danuta Dudzik, Debbie Dewaele, Laure‐Elie Carloni, Qin Tu, Yaxi Hu, Benjamin‐Florian Hempel

**Affiliations:** ^1^ Wiley Chichester UK; ^2^ CMFI Cluster of Excellence, Interfakultäres Institut für Mikrobiologie und Infektionsmedizin Tübingen Universität Tübingen Tübingen Germany; ^3^ Ion Cyclotron Resonance Program National High Magnetic Field Laboratory Tallahassee Florida USA; ^4^ Department of Marine Biology Leon H. Charney School of Marine Sciences University of Haifa Haifa Israel; ^5^ Department of Biochemistry and Molecular Genetics University of Colorado Anschutz Medical Campus Aurora Colorado USA; ^6^ Max Planck Institute of Molecular Cell Biology and Genetics Dresden Germany; ^7^ Center for Precision Medicine, Wake Forest School of Medicine Medical Center Boulevard Winston‐Salem North Carolina USA; ^8^ Department of Biopharmaceutics and Pharmacodynamics, Faculty of Pharmacy Medical University of Gdańsk Gdańsk Poland; ^9^ Clinical Release and Stability group, Analytical Development, CPDS Janssen Pharmaceutica R&D Beerse Belgium; ^10^ Advanced Material Characterization and Investigation group, Analytical Development CPDS, Janssen R&D Beerse Belgium; ^11^ College of Chemistry & Pharmacy Northwest A&F University Yangling Shaanxi P. R. China; ^12^ Food Research Division Health Canada Ottawa Ontario Canada; ^13^ BIH Center for Regenerative Therapies BCRT Charité‐Universitätsmedizin Berlin Berlin Germany

*Analytical Science Advances* is proud to announce its new Associate Editorial Board comprised of Early Career Researchers (ECRs) representing a range of sub‐disciplines across the analytical sciences. Chaired by **Benjamin‐Florian Hempel**, the group—including **Daniel Petras**, University of Tübingen; **Lissa Anderson**, National High Magnetic Field Laboratory; **Tal Luzzatto Knaan**, University of Haifa; **Anthony Saviola**, University of Colorado Anschutz Medical Campus; **Ignacy Rzagalinski**, Max Planck Institute of Molecular Cell Biology and Genetics; **Mohammad Sharif Khan**, Wake Forest Baptist Health; **Danuta Dudzik**, Medical University of Gdańsk; **Debbie Dewaele**, Janssen R&D; **Laure‐Elie Carloni**, Janssen R&D; **Qin Tu,** Northwest A&F University; and **Yaxi Hu**, Carleton University—aims to bring fresh perspectives to journal policy and editorial management.

As the number of doctoral degrees awarded worldwide continues to grow, it is crucial that traditional academic science and publishing business models established by senior academics evolve to meet the needs of today's ECRs. This Associate Editorial Board was established to:

- Increase interest and participation in editorial and publication‐related activities among ECRs.
- Bridge the gap between ECRs and more established generations of researchers.
- Increase the visibility of *Analytical Science Advances* among younger researchers.
- Nurture the next generation of authors and publishers by offering opportunities to influence journal policy and drive positive change.
- Learn ways in which scientific publishing should adapt in response to the experiences and practices of ECRs.
- Create opportunities for publishers (e.g., editorial board members, associate editors and editors‐in‐chief, etc.) to engage with ECRs at meetings and conferences.
- Support and promote open research practices and access.

Each Associate Editorial Board member, selected through a nomination process, is expected to serve a 2‐year term at which time the appointment may be extended to an additional 2 years contingent on desire to continue and review of contributions by other Associate Editorial Board members. Associate Editorial Board members will meet regularly for social think tanks with the Managing Editor, the Editors‐in‐Chief and other members of the Editorial Board to share views of the publishing process, develop tools and services that better support ECRs and evaluate selectivity criteria to address biases. All members will also interact through a quarterly meeting to ensure a continuous and dynamic dialogue aimed at determining how to better serve our authors and readers.

Going forward, *Analytical Science Advances* will adopt a three‐tiered Editorial Board structure:

1. The Editors (those individuals overseeing the handling of articles);
2. The Advisory Board and
3. The Associate Editorial Board, for whom the criteria have been expressed above.

No doubt that the new Associate Editorial Board will do its best to join the Editors and the Advisory Board in serving the broad and diverse community of analytical scientists.^1^

***

A note from the Associate editorial board chair: Benjamin‐Florian Hempel:

On behalf of the newly formed Associate Editorial Board, I would like to take the opportunity to thank Paul Trevorrow, Executive Journals Editor, Wiley for the fascinating opportunity and his enthusiasm to integrate ECRs in the world of journal policy and editorial management. This Associate Board would not exist without his unique kind to inspire young people to new adventures and his unlimited support. Analytical Science Advances debuted in 2020 and is still an emergent journal that started to cover fundamental and applied aspects of analytical sciences. We, as a highly motivated and dynamic team of ECRs in different analytical disciplines, already came up with a number of inspiring ideas and suggestions to improve the impact of the journal and allow for an enhanced researcher experience of Analytical Science Advances. Special issues, feature articles and perspectives with and from ECRs are at the very top of the to‐do list, as well as web improvement projects, social media channels and many more.

As Chair of this talented and outstanding Associate Editorial team, I feel deeply honored and already looking forward to the next meetings, hopefully in‐person again, to exchange many more ideas.

***

A note from Associate editor board member, Mohammad Sharif Khan:

Analytical science is a field that has been integrated into so many new and expanding areas that a cutting‐edge journal for related articles is required. *Analytical Science Advances* is a timely initiative. I am thrilled and delighted to be a part of this team. The association board is a new idea that nurtures the noble concept and the idea of a publication system by involving the new generation of researchers at the publishing house. Thank you to Journal's management and the publication team for their hard work. I can assure you that our authors will be glad when they submit their next exciting work to *Analytical Science Advances*.

***

A note from Associate editor board member, Tal Luzzatto Knaan:

With the advances in technology and data analysis tools, the field of Analytical Sciences is taking on a central role in multidisciplinary research, expanding the view on many scientific questions. This pioneering initiative of Analytical Science Advances to integrate ECRs in its editorial management is truly an amazing opportunity to engage and inspire young scientists in leading editorial roles and have their impact. Committed to the journals’ vision as a platform for the frontline of Analytical Science, I am honored and excited to be a part of this team.

***

A note from Associate editor board member, Lissa Anderson:

In my role as staff at a national user facility, I have had the opportunity to assist several dozens of users spanning multiple disciplines and career stages, from the US and abroad, with their analytical chemistry experiments. The users I interact with the most are the early career researchers who are in the ‘trenches’ of the laboratory performing the experiments alongside me. A major takeaway of my experiences aiding users through the publication process is that success in publication is often buried in a ‘hidden curriculum’ that can be taught through effective mentorship or that must be learned (slowly and sometimes painfully) through autodidactic methods. The educational experiences and resources available to graduate students and Postdocs vary wildly. Since the senior scientific generation are the principle gatekeepers of research dissemination, the disconnect between the ECR experience and that of the established scientist should be at the forefront of every publisher's mind at all stages of the publication process. I am encouraged by the creation of this ECR Associate Editorial Board, and grateful for the opportunity to help Analytical Science Advances foster a publication ecosystem that supports authors of diverse backgrounds to publish original, high‐quality research.
